# Toward precision in simulation of paediatric mitral valve repair using patient-specific fluid–structure interaction modelling

**DOI:** 10.1093/ehjimp/qyag103

**Published:** 2026-06-01

**Authors:** Lea Christierson, Johan Revstedt, Alice Pozza, Andreea Dragulescu, Conall Morgan, Osami Honjo, Luc L Mertens, Hanna Isaksson, Nina Hakacova

**Affiliations:** Department of Clinical Sciences, Pediatric Heart Center, Skåne University Hospital, Lund University, Lasarettgatan 42a, Lund, Sweden; Department of Biomedical Engineering, Lund University, Ole Römers väg 3, 223 63 Lund, Sweden; Department of Energy Science, Lund University, Klas Anshelms väg 4, 223 63 Lund, Sweden; Division of Cardiology, The Labatt Family Heart Centre, The Hospital for Sick Children, University of Toronto, Toronto, ON, Canada; Pediatric Cardiology Unit, Department of Women’s and Children’s Health, University of Padua, Padua, Italy; Division of Cardiology, The Labatt Family Heart Centre, The Hospital for Sick Children, University of Toronto, Toronto, ON, Canada; Division of Cardiology, The Labatt Family Heart Centre, The Hospital for Sick Children, University of Toronto, Toronto, ON, Canada; Division of Cardiovascular Surgery, The Hospital for Sick Children, Toronto, Canada; Division of Cardiology, The Labatt Family Heart Centre, The Hospital for Sick Children, University of Toronto, Toronto, ON, Canada; Department of Pediatrics, University of Toronto, Toronto, Canada; Department of Biomedical Engineering, Lund University, Ole Römers väg 3, 223 63 Lund, Sweden; Department of Clinical Sciences, Pediatric Heart Center, Skåne University Hospital, Lund University, Lasarettgatan 42a, Lund, Sweden; Division of Cardiology, The Labatt Family Heart Centre, The Hospital for Sick Children, University of Toronto, Toronto, ON, Canada

**Keywords:** mitral regurgitation, fluid–structure interaction, patient-specific, simulation, haemodynamics, valve function

## Abstract

**Aims:**

Assessment of mitral valve (MV) function and haemodynamics is essential for optimizing surgical repair in children with mitral regurgitation. Patient-specific fluid–structure interaction (FSI) modelling can capture the complex interplay between valvular mechanics and blood flow. In this study, we apply a patient-specific FSI framework to evaluate MV function and haemodynamics in paediatric patients before and after surgery.

**Methods and results:**

Seven paediatric patients with mitral regurgitation were analysed (age range: 2–17 years; median: 6 years; 57% female). Patient-specific MV apparatus geometries were segmented from pre- and postoperative 3D echocardiograms. Flow boundary conditions were derived from left ventricular volume measurements. Valve dynamics and haemodynamics were simulated using the FSI framework. Model performance was evaluated against echocardiographic data, pre- and postoperatively. The FSI model reproduced the angle of the regurgitant jet. Preoperatively, the regurgitation grade matched echocardiographic assessment in six of seven patients, and postoperatively in four of seven. The site of regurgitation was correctly identified in six of seven patients, pre- and postoperatively. The model reproduced the observed intraventricular flow patterns in most patients, and the simulated transvalvular pressure gradients agreed with Doppler measurements (mean difference: 0.38 ± 1.57 mmHg preoperatively, –0.42 ± 3.26 mmHg postoperatively).

**Conclusion:**

The proposed FSI framework captured MV function, haemodynamics, and disease-specific features in paediatric patients pre- and postoperatively, based on evaluation in one of the largest cohorts for the field. This computational framework has the potential to enable predictive simulations that could support surgical planning in the future and improve repair outcomes in children.

## Graphical Abstract

**Graphical Abstract:**
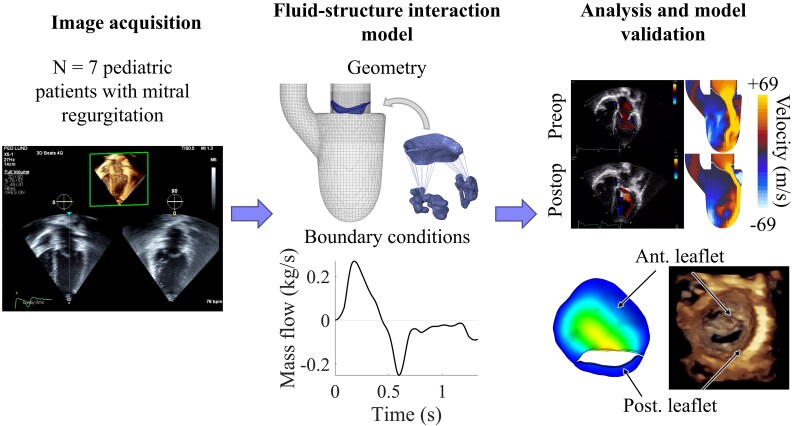

## Introduction

Moderate to severe mitral valve regurgitation in children is challenging to treat and remains a leading cause of heart failure.^1^ Surgical decision-making is complex, as valve repair can lead to suboptimal outcomes with residual stenosis and/or regurgitation, frequently requiring reintervention.^2–6^ Predicting potentially unsuccessful valve function and haemodynamic outcomes of valve repair remains difficult using conventional imaging alone. While echocardiography provides essential real-time anatomical and functional information, it offers limited insight into the future function of the repaired valve. This inability to predict postoperative valve function complicates management and may expose children to repeat surgeries or prosthetic replacement.^6^ Tools are needed to identify the mechanisms of regurgitation and predict the impact of different repair strategies, thus enhancing surgical planning, individualizing treatment, and reducing the risk of unsuccessful repair.

Patient-specific computational models based on fluid–structure interaction (FSI) provide a promising tool for assessing valve function and exploring surgical scenarios preoperatively. Our group has developed and experimentally validated such a framework^7,8^ and validated it in healthy volunteers based on haemodynamic parameters used for mitral valve assessment,^9^ supporting its clinical applicability in both adults and children. Most prior FSI studies address adult mitral valve disease lack validation against *in vivo* data,^10–13^ rely on normal reference values from the literature,^11,13–16^ or use single-patient cohorts.^14–16^ Thus, patient-specific validation and validation in larger patient cohorts remain limited, which restricts the interpretation and clinical reliability of the simulation results. Further, most previous studies focus solely on the preoperative condition,^10,11,15,17,18^ and few investigations have modelled the postoperative valve configurations,^17,19^ of which none have validated them against *in vivo* postoperative data. Studies of paediatric mitral valves are even scarcer, limited to finite element analyses^19,20^ without haemodynamic integration, which is essential for clinical evaluation of the valve function.

This study presents the feasibility of a patient-specific FSI framework to model paediatric mitral regurgitation before and after surgery. We evaluated the simulated systolic and diastolic valve function against echocardiographic data from seven patients, representing one of the biggest patient cohorts published so far in the field of patient-specific FSI simulations. Our model incorporates patient-specific haemodynamics and patient-specific valve geometries to represent the *in vivo* conditions. Our goal is to provide a novel tool for patient-specific evaluation that may ultimately guide clinical decision-making and gain insight into the mechanisms behind the regurgitation.

## Methods

**Figure 1:**
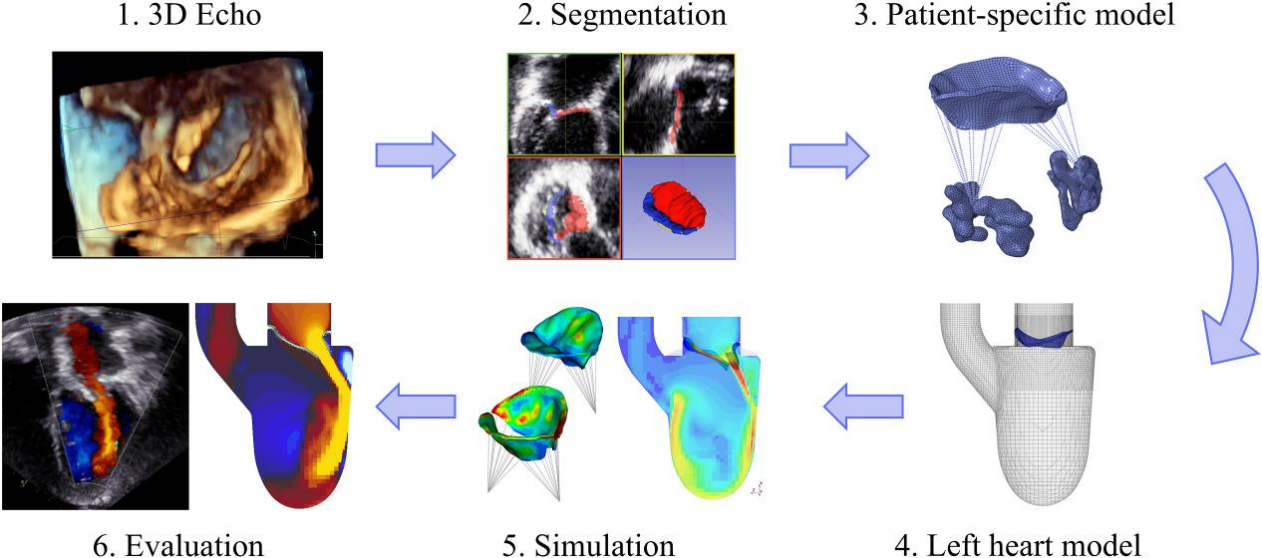
A schematic overview. The framework for patient-specific FSI simulations of mitral valve regurgitation is illustrated in six steps: (1) The valve was imaged with 3D echocardiography (echo), from which (2) the valve was segmented, and (3) a patient-specific valve geometry could be constructed. (4) The valve apparatus was inserted into a left-heart model, and( 5) simulated with patient-specific mass flow. (6) Finally, the results were evaluated against echocardiography.

In this feasibility study, we applied our previously validated simulation framework for modelling mitral valve function in healthy volunteers^9^ to model paediatric patients with regurgitant mitral valves, both pre- and postoperatively (*Figure 1*). Patient-specific valve geometries were reconstructed from 3D echocardiography, meshed, and integrated into a left-heart model with attached chordae tendineae. Patient-specific flow boundary conditions, derived from echocardiographic data, were used to drive haemodynamics.

Each valve was simulated in its pathological and repaired configurations. The model performance was evaluated against echocardiographic data in both conditions, focusing on systolic and diastolic valve function.

### Study population

Paediatric patients with clinically significant mitral regurgitation who had undergone surgical repair were retrospectively recruited at the Hospital for Sick Children, Toronto. Four patients were excluded due to incomplete pre- or postoperative echocardiographic datasets, and one due to concomitant severe aortic regurgitation, leaving seven patients (aged 2–17 years; median 6 years; 57% female) for analysis (*Tables 1–2*). The study was approved by the SickKids Research Ethics Board (REB: 81838) and complies with the Helsinki Declaration.

**Table 1:** Patient demographics

					Preoperatively				Postoperatively			
Patient	Sex	Age (years)	Weight (kg)	Height (cm)	Pre-op exam. (weeks)	BPM	EDV (ml)	ESV (ml)	Post-op exam. (weeks)	BPM	EDV (ml)	ESV (ml)
**1**	M	11	28.7	144	25	88	180	71	0.4	106	125	74
**2**	F	12	31	145	2	81	100	50	8	75	80	38
**3**	M	7	22.7	120	3	126	134	50	0.7	125	83	51
**4**	M	5	21.2	107	2	149	48	11	0.3	98	46	23
**5**	F	2	14.6	99	17	80	52	21	0.3	105	56	26
**6**	F	2	10	87	0.1	153	96	59	0.9	109	116	74
**7**	F	17	49	155	0.1	69	200	86	0.6	71	168	112

Patient information reported pre- and postoperatively. The dates of the pre- and postoperative examinations (where the data used in this study was acquired) are indicated in terms of weeks before and after surgery. BPM = beats per minute; EDV = end-diastolic volume; ESV = end-systolic volume; F = female; M = male; sex = sex assigned at birth.

**Table 2:** Anamnesis for the patients

Patient	MR grade pre-op	Diagnosis	Mitral valve repair	MR grade post-op
**1**	Severe	Severe mitral regurgitation with pseudo cleft at PML	Release of secondary chordae at PML, skeletonization of papillary muscle, closure of PML pseudo cleft with annuloplasty band	Mild
**2**	Severe	Severe mitral regurgitation due to rheumatic fever	Neochordae to AML, annuloplasty band, patch enlargement of PML	Trivial
**3**	Moderate	Mitral regurgitation due to rheumatic fever and severe progressive LV/LA dilatation	Bilateral partial annuloplasties, neochordae to AML, patch enlargement of PLM	Mild
**4**	Moderate	Mitral regurgitation due to dysplastic MV with AML prolapse, tricuspid regurgitation, congestive heart failure	PML patch enlargement, neochordae to AML, posterior annuloplasty, delamination of PML, mobilization of papillary muscles	Trivial
**5**	Moderate	Mitral regurgitation with AML prolapse	Neochordae to AML, annuloplasties to lateral and medial commissures	Trivial
**6**	Severe	Severe mitral regurgitation due to dilated cardiomyopathy	Posterior annuloplasty band, closure of posterior cleft, valvuloplasties at posteromedial commissure	Moderate
**7**	Severe	Severe mitral regurgitation due to cleft in AML	Cleft closure with pericardial patch, annuloplasty band	Trivial

Description of the diagnosis, regurgitation grade, and the mitral valve repair strategy for each patient. AML = anterior mitral leaflet; MR grade pre-/post-op = mitral regurgitation grade pre-/postoperatively; MV = mitral valve; PML = posterior mitral leaflet; LV = left ventricle; LA = left atrium.

### Image acquisition and segmentation

Pre- and postoperative echocardiographic data were obtained from routine clinical examinations performed by experienced paediatric cardiologists using a Vivid E95 system (GE Healthcare, Vingmed Ultrasound, Horten, Norway). For simulation input, 3D zoom datasets of the mitral valve and full-volume acquisitions of the left ventricle were collected. Doppler spectral curves of the transvalvular flow were used for subsequent evaluation.

**Figure 2:**
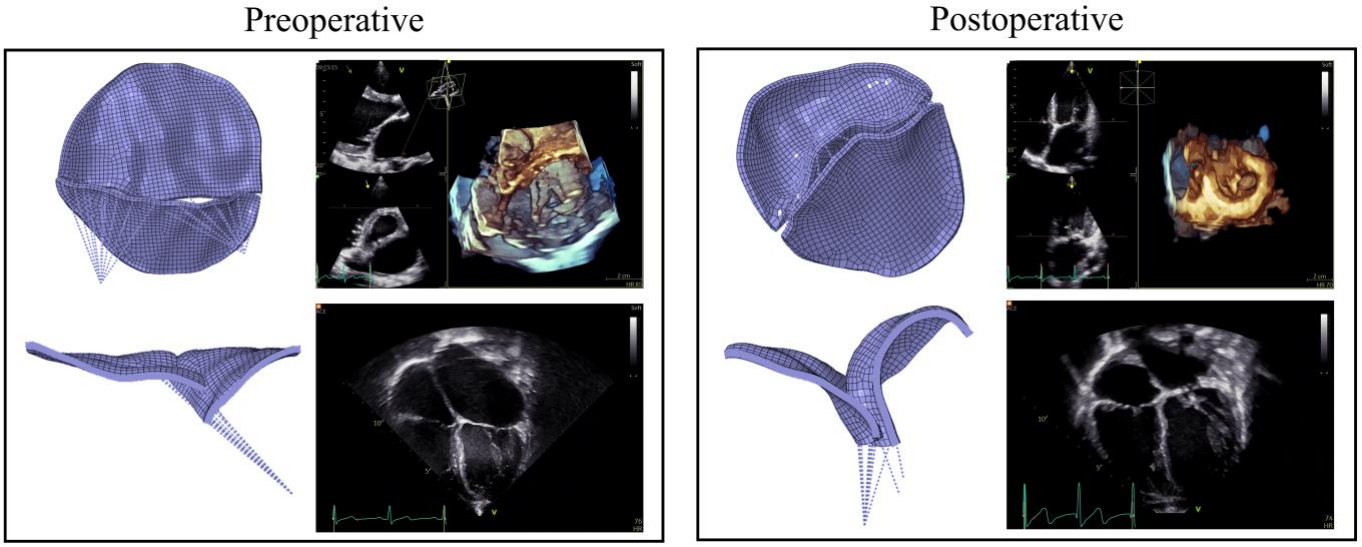
The valve segmentation. The pre- and postoperative segmentations for Patient 2 with the corresponding 3D echocardiography, at end-systole.

Patient-specific mitral valve geometries were manually segmented (3D Slicer^21^) (*Figure 2*), with careful delineation of pathological features. An inter-observer analysis of the manual segmentation showed a 3.16–4.73% difference between observers (see Supplementary Material  *A*). The papillary muscle positions were identified and segmented for accurate placement of the papillary heads. The final geometries were cross-checked against 2D echocardiographic measurements for dimensional accuracy. Patient-specific flow boundary conditions were derived from 3D echocardiograms of the left ventricle with a semi-automated segmentation approach. The end-diastolic and end-systolic volumes were verified using Simpson’s method.

### The simulation model

The simulation pipeline, developed by our group and validated experimentally^7^ and in healthy volunteers,^9^ modelled the mitral valve leaflets as hyperelastic tissue based on experimental data.^22^ Chordae tendineae were represented as tensile-only linear elastic elements attached from the leaflet free edges to the two papillary muscle heads. Leaflet contact was handled with a zero-penetration penalty-based contact algorithm.

The fluid domain encompassed the left atrium, left ventricle, and left ventricular outflow tract. The blood was modelled as laminar and as a non-Newtonian fluid using the Carreau model. Time-resolved mass flow rates, derived from ventricular volume changes, were used as boundary conditions to reproduce the patient-specific haemodynamics. To account for surgical repair, pre- and postoperative boundary conditions were applied separately based on the corresponding echocardiographic data. The aortic valve was modelled as a free-flow outlet during systole and as a closed wall during diastole.

The large mitral leaflet deformations were resolved using an overset mesh technique, circumventing the need for remeshing. Simulations were performed in *Star-CCM+* (v2022.1, Siemens Digital Industries Software, Plano, TX, USA) coupled with *Abaqus/CAE* (v2020, Dassault Systèmes Simulia Corp., Johnston, RI, USA) using a transient time step of 0.4 ms. Further methodological details are provided in Christierson *et al*.^9^

### Investigated parameters and statistical analysis

#### Systolic valve function

The simulated atrial regurgitant jet was evaluated qualitatively with colour Doppler echocardiography across patients to identify the angle of the regurgitant jet.

The regurgitation grade was visually assessed by two independent observers: (i) the senior cardiologist reviewing the echocardiograms for clinical purposes, and (ii) a senior cardiologist after inclusion into the study (N.H.). The visual assessment was performed in both echocardiography and simulations using two-chamber, four-chamber, and short-axis views.^23,24^ The grading was performed blindly, and the simulation results were compared with the echocardiographic findings in a binary agree/disagree fashion.

The site of regurgitation was determined in the segmented valves and in the echocardiograms by an experienced paediatric cardiologist (N.H.). The assessment was performed blinded on pre- and postoperative data for all patients. The location of regurgitation was defined in terms of the affected leaflet segments in the simulation results as well as the echocardiograms. The leaflet segments were defined according to Carpentier/ASE nomenclature (A1-A3 for the anterior and P1-P3 for the posterior leaflet).^25^ The simulated location of regurgitation was compared with the echocardiographic findings in binary agreement terms.

#### Diastolic valve function

The simulated left ventricular haemodynamics were evaluated qualitatively via frame-by-frame comparison with colour Doppler echocardiography. The ventricular filling was investigated across patients pre- and postoperatively to identify different flow patterns.

The simulated transvalvular flow was quantitatively compared with Doppler spectral measurements, which were averaged over three cardiac cycles to reduce measurement noise and variability. The transvalvular pressure gradient, defined as the pressure difference between the atrial and ventricular sides of the mitral valve, was calculated from the simulated flow velocity using the simplified Bernoulli equation^26^ in accordance with the corresponding Doppler measurements. Agreement between the simulated and measured gradients, pre- and postoperatively, was reported as mean difference ± standard deviation and further evaluated using Bland–Altman analysis.^27^

## Results

### Systolic mitral valve function

**Figure 3:**
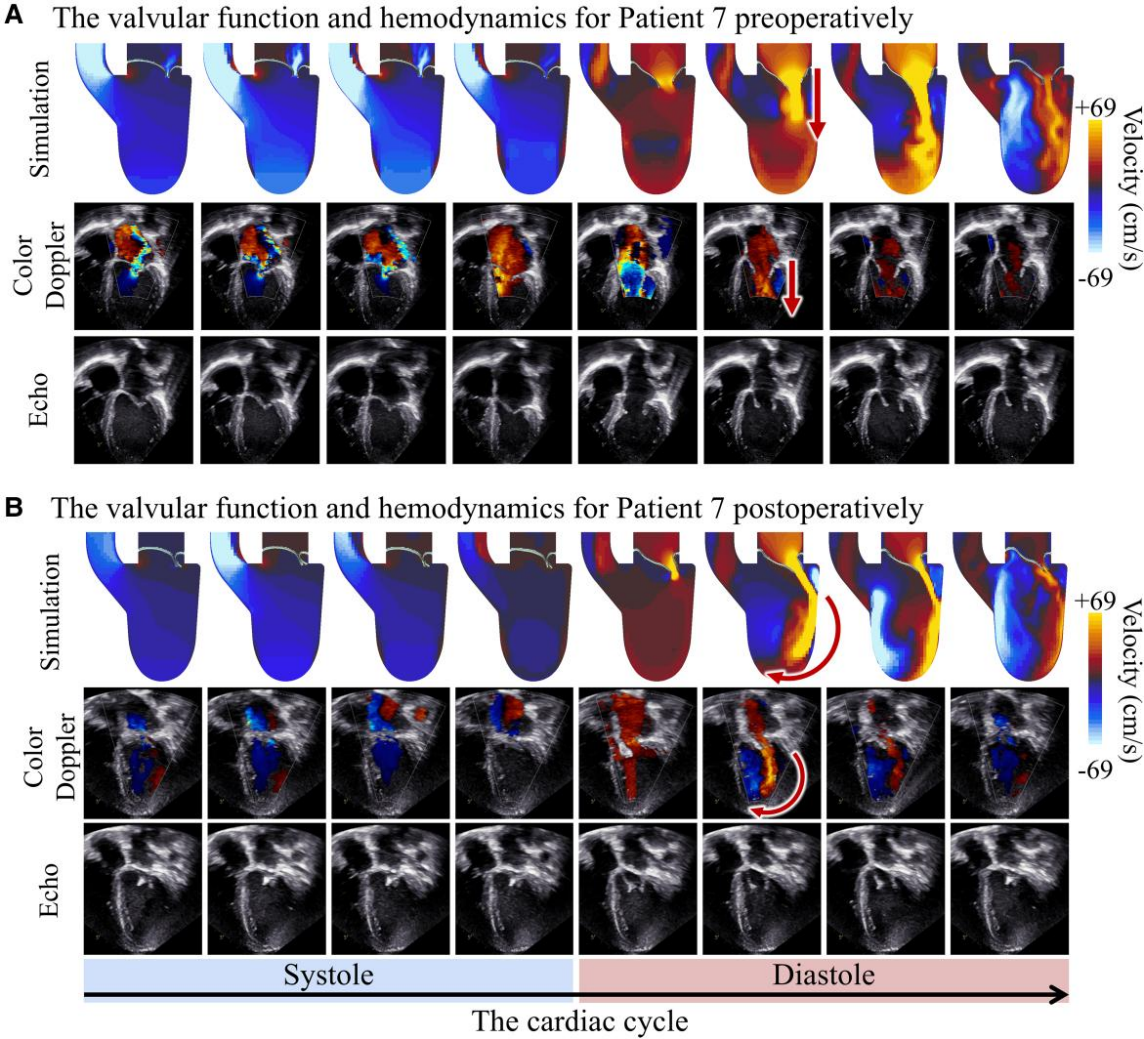
The valve function and haemodynamics. Snapshots of (A) preoperative and (B) postoperative valvular function from simulations compared with echocardiography, with and without colour Doppler. Patient 7 is shown as a representative case with preoperative severe mitral regurgitation due to a central anterior leaflet cleft and restricted posterior leaflet. Surgical repair included cleft closure with a pericardial patch and resection of secondary posterior chordae to improve mobility. Simulation and Doppler velocities are displayed using the same colour scale. The arrows indicate the inflow creating different intraventricular flow patterns. The corresponding video can be found in Supplementary data online, Material  *B*.

During systole, the angle of the regurgitant jet into the left atrium was consistently captured across all seven patients (*Figure 3A*). Postoperatively, the model reproduced the residual regurgitant jets in three of four patients with visible regurgitant jets.

**Figure 4:**
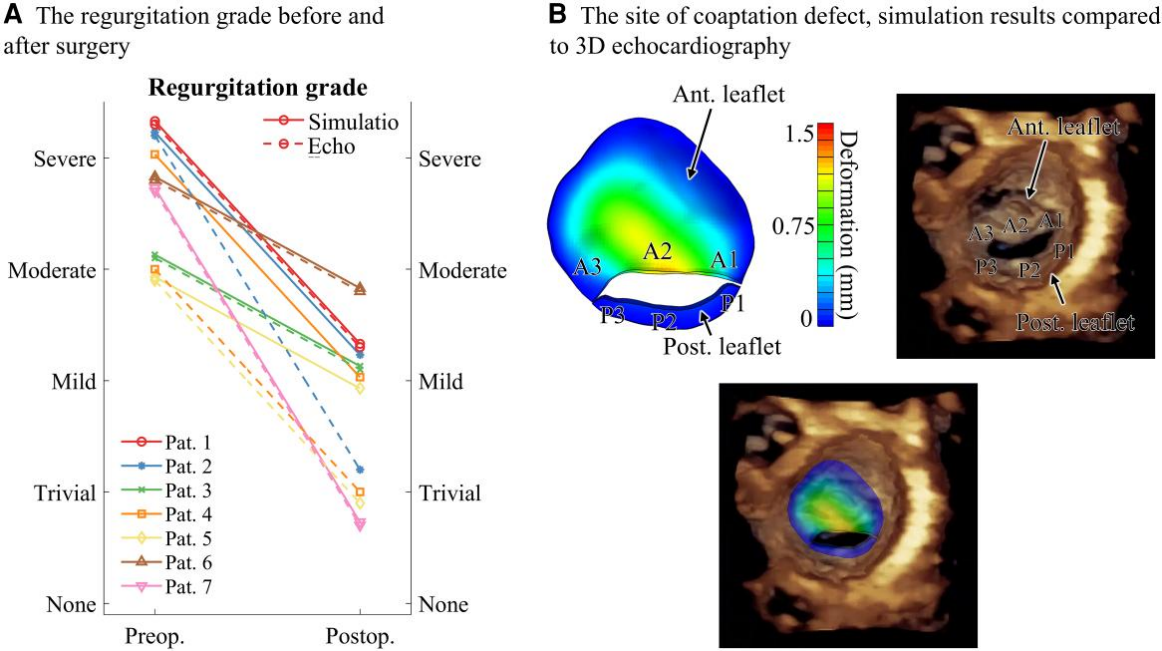
The systolic valve function. (*A*) Comparison of simulated (solid lines) and echocardiographic (dashed lines) regurgitation grades pre- and postoperatively. (*B*) The simulated and imaged preoperative coaptation defect in Patient 4 is visualized and compared at peak systole. The valve is viewed from the atrial side preoperatively, with the Carpentier/ASE segments: A1-A3 for the anterior (ant) and P1-P3 for the posterior (post) leaflet marked out.

Preoperatively, the simulated and echocardiographic regurgitation grades agreed in six of seven patients (*Figure 4A*). In one case (Patient 4), the model graded regurgitation as severe compared with moderate by echocardiography. Postoperatively, agreement was observed in four of seven patients. In the remaining three (Patients 2, 4, and 5), the model slightly overestimated regurgitation, simulating mild rather than trivial regurgitation.

The simulated location of coaptation defects matched echocardiography in six of seven patients preoperatively (*Figure 4B*), differing only in Patient 1 (simulation: A2/P2; echocardiography: A1/P1) (*Table 3*). Postoperatively, agreement remained in six of seven patients, with one mismatch (Patient 5: simulation A1/P1; echocardiography A2/P2) (*Table 4*).

**Table 3:** Site of regurgitation

	Preoperatively		Postoperatively	
Patient	Simulation	Echocardiography	Simulation	Echocardiography
**1**	A2/P2	A1/P1	A1/P1	A1/P1
**2**	A1/P1	A1/P1	A1/P1	A1/P1
**3**	A3/P3	A3/P3	Whole commissure	Whole commissure
**4**	Whole commissure	Whole commissure	A3/P3	A3/P3
**5**	A3/P3	A3/P3	A1/P1	A2/P2
**6**	A2/P2	A2/P2	Whole commissure	Whole commissure
**7**	Whole commissure and cleft	Whole commissure and cleft	A3/P3	A3/P3

The site of regurgitation in the simulations and echocardiography, based on the Carpentier/ASE segments (as defined in *Figure 4B*).

**Table 4:** Types of left ventricular flow

	Preoperatively		Postoperatively	
Patient	Simulation	Echocardiography	Simulation	Echocardiography
**1**	Straight	Straight	Clockwise	Clockwise
**2**	Straight	Straight	Straight	Straight
**3**	Clockwise	Clockwise	Clockwise	Clockwise
**4**	Clockwise	Clockwise	Straight	Straight
**5**	Straight	Clockwise	Clockwise	Clockwise
**6**	Clockwise	Clockwise	Clockwise	Clockwise
**7**	Clockwise	Straight	Clockwise	Clockwise

The type of left ventricular flow pattern observed in the simulation results, as well as in the patients’ echocardiography. The ‘straight’ and ‘clockwise’ terms refer to the flow obtained in the left ventricle based on the inflow jet from the mitral valve.

### Diastolic mitral valve function

The model accurately reproduced the main haemodynamics behaviour across the valve in both the pre- and postoperative conditions when qualitatively compared with colour Doppler imaging (*Figure 3*). Two different ventricular flow patterns were observed: where the inflow jet caused the ventricular flow to rotate clockwise in the ventricle, and where the inflow jet remained straight, dissipating into the ventricle (*Table 4*). Both ventricular flow behaviours were accurately reproduced by the FSI model, capturing the flow in five of seven patients preoperatively, and in all patients postoperatively (*Figure 3*). A representative example, shown in *Figure 3*, illustrates the close agreement between the simulated and echocardiographic ventricular flow. The diastolic ventricular flow was particularly well reproduced in the later systolic frames of both simulation and echocardiography. A video of the valve, pre- and postoperatively, as simulated by the model and compared with colour Doppler echocardiography, can be seen in Supplementary data online, Material  *B*.

**Figure 5:**
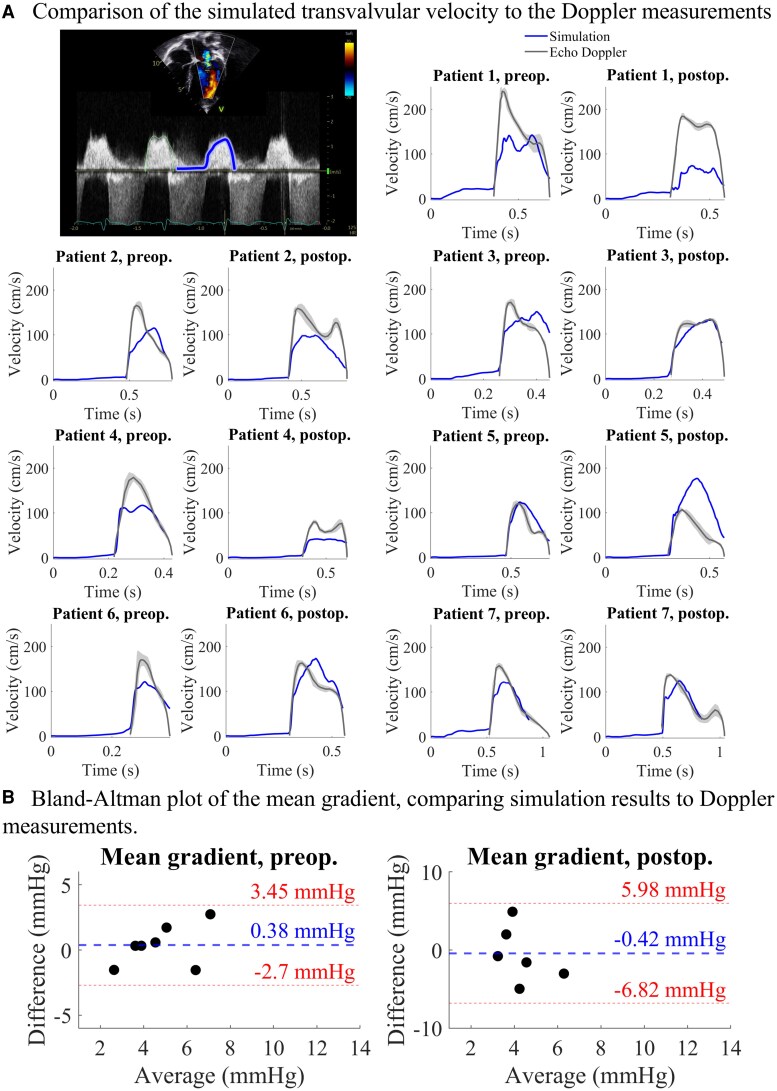
The diastolic valve function. (*A*) Transvalvular flow pre- and postoperatively, comparing simulations (blue) with continuous wave Doppler measurements (black). Postoperative results for Patient 3 are overlaid on the Doppler spectral curve for illustration. (*B*) Bland–Altman plot of mean diastolic pressure gradient, pre- and postoperatively. Differences between simulated and Doppler measurements are plotted against the average of both datasets. Blue dotted line, mean difference; red dotted lines, 95% limits of agreement.

The simulated transvalvular velocities were compared with continuous-wave Doppler measurements for all patients (*Figure 5A*). Preoperatively, the mean difference between simulated and Doppler-derived mean transmitral gradients was 0.38 ± 1.57 mmHg, with 95% limits of agreement from −2.70 to 3.45 mmHg (*Figure 5B*). Postoperatively, the mean difference was −0.42 ± 3.26 mmHg, with 95% limits of agreement from −6.82 to 5.98 mmHg (*Figure 5B*).

## Discussion

In this study, we present a patient-specific FSI framework capable of modelling paediatric mitral valve regurgitation under pre- and postoperative conditions. The model utilizes standard 2D and 3D echocardiographic data to reconstruct patient-specific mitral valve geometries, including the subvalvular apparatus, and patient-specific flows. The framework accurately captured the overall valve dynamics and haemodynamics, reproducing quantitative measures such as mean transvalvular pressure gradient, regurgitation grade, and regurgitation site when compared with echocardiography. The novelty of this study lies in its application to paediatric mitral valves, the incorporation of patient-specific valve segmentation, and the comprehensive evaluation of systolic and diastolic valve function in pre- and postoperative conditions based on *in vivo* data in a large cohort, for the field of FSI modelling. By extending beyond theoretical modelling and incorporating patient-specific evaluation, this feasibility study shows that our approach can capture the valve function and disease-specific features pre- and postoperatively. This represents a step towards predictive simulations that, in the future, hold the potential to inform preoperative planning by simulating the postoperative result.

### Systolic mitral valve function

The angle of the regurgitant jet has diagnostic value, as it reflects the underlying mechanism of regurgitation, a feature that was captured by our model. Primary regurgitation, associated with leaflet prolapse, typically produces an angled jet directed along the valve leaflet (eccentric jet), whereas secondary regurgitation, resulting from ventricular dilation, generates a centrally directed jet directed into the atrium (concentric jet).^28^

Preoperatively, the simulated regurgitation grade aligned with echocardiography in most patients. Postoperatively, discrepancies in differentiating lower regurgitation grades were observed (e.g. trivial vs. mild), implying that these are more challenging to resolve by our model. However, both trivial and mild regurgitation grades are acceptable postoperative results. The site of regurgitation matched in all patients except one, indicating that although the segmented valve provides a good representation of the valve, the load from the blood flow affects where the regurgitation occurs. In this study, assessment of regurgitation grade and site was performed by an experienced paediatric cardiologist based on visual assessment. Although this corresponds to clinical practice, it can pose a limitation; thus, future development of the computational framework entails introducing quantitative measures of the regurgitant volume fraction.

To our knowledge, this is the first study to directly compare simulated and echocardiographic assessments of both regurgitation site and severity in pre- and postoperative configurations. Previous FSI studies have not combined these aspects,^15,29,30^ but have compared either the coaptation area,^19^ the regurgitant volume,^29^ or orifice area^31,32^ between simulated healthy and diseased adult valves. Other studies have focused on aortic valve disease,^11,33–35^ and lacked *in vivo* validation.^12,19,36^ For paediatric cohorts, finite element models have been used to assess mitral regurgitation,^32^ however, omitting the haemodynamics limits the clinical interpretation. In contrast, our model reproduced the combined grade and site of regurgitation, as well as the transmitral flow, compared against echocardiographic data from seven paediatric patients.

### Diastolic mitral valve function

The left heart haemodynamics were well reproduced, with simulated ventricular flow matching *in vivo* measurements, supporting the model’s reliability. The model captured key features of both the diseased and repaired valves, such as the clockwise rotation of the intraventricular flow. The clockwise rotation of the intraventricular flow is an indicator of improved valve–ventricle interaction that aids mitral valve closure and improves aortic outflow^37^ and has been described previously.^38^ In this study, this pattern was observed in some of the patients; however, the cohort is too small to draw any population-based conclusions. Prior studies primarily investigated left-heart haemodynamics after edge-to-edge^30,31^ or MitraClip repair^17^ in adults without validation against *in vivo* surgical data, limiting the interpretation of the model accuracy. In contrast, our study focuses on paediatric patients, a population rarely explored with FSI modelling, and evaluates simulations against echocardiographic data from seven children, enabling the investigation of patient-specific traits.

The simulated transvalvular velocities showed slightly greater deviation from Doppler data, yet the model consistently yielded postoperative reduction in velocity, consistent with decreased end-diastolic volume and mass flow following valve repair.^39^ Clinically, the mean transvalvular pressure gradient is one of the few routinely used quantitative metrics for assessing mitral valve function, with grading thresholds typically in 5 mmHg increments.^26,40,41^ In our simulations, the mean absolute error relative to Doppler-derived gradients was <1 mmHg, well within the 2.7 ± 1.1 mmHg difference reported between Doppler and catheter-based measurements.^42^ Thus, even though our observed deviations are within expected Doppler variability,^43^ which implies that the simulation model can capture the transvalvular pressure gradient well, the cohort size is too small to draw any population-based conclusions. Compared with prior studies that focused only on preoperative conditions^10,11,13,14^ or excluded flow in postoperative models,^17,19,36^ our framework advances the field by patient-specific evaluation of valve function and haemodynamics against *in vivo* data, pre- and postoperatively. Most prior work on mitral regurgitation addressed solely the systolic valve function, without investigating the diastolic function, such as the transvalvular flow,^13,15,31^ which limits the assessment of the overall valve function for the entire cardiac cycle. Our framework achieves comparable accuracy of the transmitral velocity,^38,44^ when compared against other FSI models of healthy valves, while uniquely extending this capability in this study to diseased and repaired paediatric valves, providing a clinically relevant, quantitative evaluation of surgical outcomes.

### Holistic evaluation

A comprehensive understanding of valve function requires integrated interpretation of all investigated parameters. Across the cohort, surgical repair was expected to reduce end-diastolic volume, which was reflected in the simulations by lower postoperative transmitral velocities and pressure gradients in most patients. The reduced postoperative regurgitation grades across the cohort further demonstrated improvements in valve competence, consistent with the clinically successful surgical outcomes. In addition, most patients exhibited clockwise intraventricular flow rotation postoperatively, suggesting improved valve–ventricle interaction.

Importantly, the model consistently reproduced the expected relationship between regurgitation grade, jet orientation, and regurgitation site for the underlying pathology, thereby capturing both the severity and the underlying mechanism of regurgitation. No clear association was observed between regurgitation grade or location and the resulting intraventricular flow pattern, which may reflect either inter-patient variability or the limited cohort size. Nevertheless, the combined findings demonstrate that the model provides a coherent representation of both systolic and diastolic mitral valve function in paediatric mitral regurgitation.

### Limitations

The patient-specific mass flow boundary conditions derived from ventricular volume curves were used to model ventricular contraction, while the ventricular walls and papillary muscle heads were modelled as fixed in space. Although this simplification achieved good agreement in healthy volunteers,^9^ it may be less accurate in diseased valves, where the valve–ventricle interaction plays a more significant role. Future work, incorporating anatomical motion, could clarify its influence on simulated valve mechanics.

A further limitation of this study is that the model evaluation relies in part on qualitative comparisons with echocardiographic data, including visual assessment of ventricular flow patterns and binary agreement metrics for regurgitation grade and location. However, these assessments closely reflect current clinical practice, where the initial evaluation of congenital mitral valve disease is largely based on expert echocardiographic interpretation of flow patterns, jet orientation, and regurgitation severity. Nevertheless, future work should aim to incorporate additional quantitative metrics to further strengthen the robustness of the evaluation.

## Conclusions

This feasibility study introduced a patient-specific FSI framework capable of modelling paediatric mitral valve regurgitation before and after surgical repair, using standard clinical echocardiography. The simulations reproduced the mitral valve dynamics and haemodynamics with strong agreement to echocardiographic observations, based on evaluation in seven patients, in both the pre- and postoperative configurations.

To our knowledge, this is the first fully coupled FSI framework applied to a paediatric cohort with mitral valve regurgitation and compared against both pre- and postoperative *in vivo* data. This work fills a critical gap in paediatric mitral valve modelling and establishes a foundation for capturing patient-specific variations. Ultimately, our framework has the potential to enable virtual surgical planning by simulating the postoperative valve function and haemodynamics, and thus improving the chances for positive mitral valve repair outcomes.

## Supplementary Material

qyag103_Supplementary_Data

## Data Availability

The research data provided in this study may be provided upon request by contacting the corresponding author.

## Lead author biography

Lea Christierson has a bachelor’s degree in engineering physics and a master’s degree in mechanical engineering. After her graduation, she pursued a PhD, where she immersed herself in the world of patient-specific simulations of the mitral valve using fluid–structure interaction (FSI). FSI is a powerful tool that combines computational fluid mechanics and computational solid mechanics and can therefore be used to determine how the blood flow affects the heart valve and how the heart valve affects the blood flow. She defended her thesis in November 2025 and has continued on the same path as a postdoctoral researcher.
