# Supplementary material for: Toward precision in simulation of paediatric mitral valve repair using patient-specific fluid–structure interaction modelling
Source: Eur Heart J Imaging Methods Pract. 2026 Jun 1;4(1):qyag103. doi: 10.1093/ehjimp/qyag103 (PMC13274469; doi:10.1093/ehjimp/qyag103)
Supplement: qyag103_Supplementary_Data [file qyag103_supplementary_data.zip › Christierson_et_al_Modeling_Mitral_Regurg_Supp_Mat.pdf]

SUPPLEMENTAL MATERIAL

Toward Precision in Simulation of Pediatric Mitral Valve Repair Using Patient-Specific Fluid-Structure Interaction Modeling

Authors: Lea Christierson, Johan Revstedt, Alice Pozza, Andreea Dragulescu, Conall Morgan, Osami Honjo, Luc Mertens, Hanna Isaksson, Nina Hakacova

Supplementary material A: Inter-observer sensitivity analysis

An inter-observer sensitivity analysis was performed to investigate the reproducibility of the manual segmentation. Two different observers manually segmented pediatric heart valves, blinded to the patient's name and pseudonym. The segmented valves between observers were compared based on the anterior-posterior (A-P) diameter and the anterolateral-posteromedial (AL-PM) diameter (Fig. S1). The A-P diameter averaged 24.9 mm (range: 16.9-29.8 mm), and the AL-PM diameter averaged 26.2 mm (range: 20.4-30.9 mm). Comparisons showed average differences in the A-P diameter of 4.73 % and 3.16% for the AL-PM diameter between the two observers. These variabilities are within the widely considered clinically acceptable range of up to 10% [1].

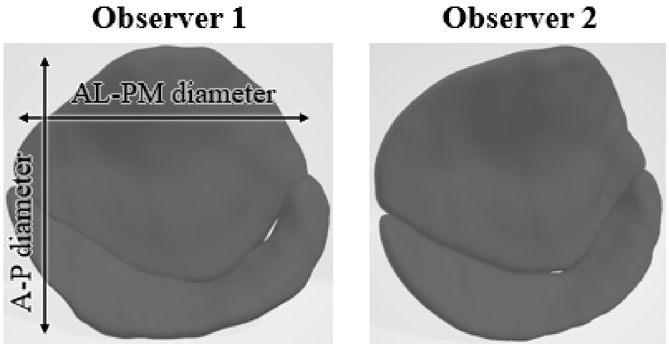

Figure S1: *An inter-observer sensitivity analysis of the segmented heart valves was conducted. To exemplify the difference between observers, the segmented valves of Patient 1, post-operatively, are shown for each observer.*

Supplementary material B: Video of valve function

A video of the simulated Patient 7 and the corresponding color Doppler cine, compared before and after surgery, can be found in the online material (Fig. S2).

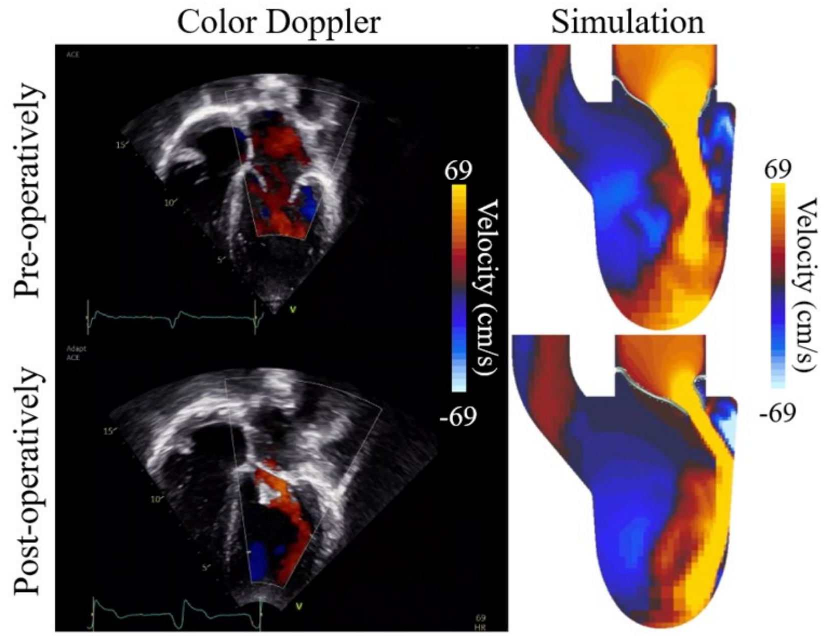

Figure S2: Thumbnail of the video material for Patient 7, comparing simulation results to color Doppler cine pre- and post-operatively.

References

[1] V. Mor-Avi *et al.*, "Real-Time 3-Dimensional Echocardiographic Quantification of Left Ventricular Volumes Multicenter Study for Validation With Magnetic Resonance Imaging and Investigation of Sources of Error," 2008.
